# The Rural Household Multiple Indicator Survey, data from 13,310 farm households in 21 countries

**DOI:** 10.1038/s41597-020-0388-8

**Published:** 2020-02-11

**Authors:** Mark van Wijk, James Hammond, Leo Gorman, Sam Adams, Augustine Ayantunde, David Baines, Adrian Bolliger, Caroline Bosire, Pietro Carpena, Sabrina Chesterman, Amon Chinyophiro, Happy Daudi, Paul Dontsop, Sabine Douxchamps, Willy Desire Emera, Simon Fraval, Steven Fonte, Lyda Hok, Henry Kiara, Esther Kihoro, Luke Korir, Christine Lamanna, Chau T. M. Long, Godfrey Manyawu, Zia Mehrabi, Dejene K. Mengistu, Leida Mercado, Katherin Meza, Vesalio Mora, Jacob Mutemi, Mary Ng’endo, Paulin Njingulula, Chris Okafor, Tim Pagella, Phonepaseuth Phengsavanh, James Rao, Randall Ritzema, Todd S. Rosenstock, Tom Skirrow, Jonathan Steinke, Clare Stirling, Jose Gabriel Suchini, Nils Teufel, Peter Thorne, Steven Vanek, Jacob van Etten, Bernard Vanlauwe, Jannike Wichern, Viviane Yameogo

**Affiliations:** 1 International Livestock Research Institute (ILRI), PO Box 30709, Nairobi, 00100 Kenya; 2 Alan Turing Institute, British Library, 96 Euston Road, London, NW1 2DB England; 3 TreeAID, Brunswick Court, Brunswick Square, Bristol, BS2 8PE UK; 4 International Centre for Tropical Agriculture (CIAT), Asia Regional Office, Hanoi, Vietnam; 5 Hanns R. Neumann Stiftung, Kota Liwa, 34812 Lampung Barat, Indonesia; 6 World Agroforestry (ICRAF), PO Box 30677-00100, UN Avenue, Nairobi, Kenya; 7 NASFAM, African Unity Avenue, Lilongwe, Malawi; 8 Tanzania Agricultural Research Institute – Naliendele, Mtwara, Tanzania; 9 International Institute of Tropical Agriculture (IITA), P.O. Box 30772-00100, Nairobi, Kenya; 10 Animal Production Systems Group, Wageningen University & Research, P.O. Box 338, Wageningen, 6700 AH The Netherlands; 11 Department of Soil and Crop Sciences, Colorado State University, Fort Collins, CO 80523 USA; 12 Faculty of Agronomy and Centre of Excellence on Sustainable Agricultural Intensification and Nutrition, Royal University of Agriculture, Phnom Penh, 12401 Cambodia; 13 Western Highlands Agriculture and Forestry Science Institute (WASI), Buon Ma Thuot, Vietnam; 14 The UBC School of Public Policy and Global Affairs, University of British Columbia, Vancouver, British Columbia V6T 1Z2 Canada; 15 Bioversity International, Rome, Italy; 16 CATIE - Centro Agronómico Tropical de Investigación y Enseñanza, Turrialba, Costa Rica; 17 Grupo Yanapai, Calle Tripoli 365, Miraflores, Lima Peru; 18 Ministerio de Agricultura y Ganadería, Siquirres, Costa Rica; 19 Lutheran World Relief, Nairobi, Kenya; 20 School of Natural Sciences, Bangor University, Bangor, UK; 21 National Agriculture and Forestry Research Institute (NAFRI), Vientiane, Lao PDR Laos; 22 Olivet Nazarene University, One University Avenue, Bourbonnais, IL60914 USA; 23 International Maize and Wheat Improvement Centre (CIMMYT), Sustainable Intensification Program, Mexico, DF Mexico; 24 Plant Production Systems group, Wageningen University, P.O. Box 338, 6700 AH Wageningen, The Netherlands

**Keywords:** Agriculture, Environmental social sciences

## Abstract

The Rural Household Multiple Indicator Survey (RHoMIS) is a standardized farm household survey approach which collects information on 758 variables covering household demographics, farm area, crops grown and their production, livestock holdings and their production, agricultural product use and variables underlying standard socio-economic and food security indicators such as the Probability of Poverty Index, the Household Food Insecurity Access Scale, and household dietary diversity. These variables are used to quantify more than 40 different indicators on farm and household characteristics, welfare, productivity, and economic performance. Between 2015 and the beginning of 2018, the survey instrument was applied in 21 countries in Central America, sub-Saharan Africa and Asia. The data presented here include the raw survey response data, the indicator calculation code, and the resulting indicator values. These data can be used to quantify on- and off-farm pathways to food security, diverse diets, and changes in poverty for rural smallholder farm households.

## Abstract (summary)

**Table:** 

Measurement(s)	Demographics • cultivated environment • Household Environment • economic performance
Technology Type(s)	Survey
Factor Type(s)	country • year
Sample Characteristic - Organism	Homo sapiens
Sample Characteristic - Environment	farm
Sample Characteristic - Location	Central America • Sub-Saharan Africa • Asia

Machine-accessible metadata file describing the reported data: 10.6084/m9.figshare.11637786

## Background & Summary

Agriculture is the most important livelihood option for most rural households in low- and middle-income countries^1^. Smallholder farm households in these locations produce food not only for themselves, but, in many countries, produce the majority of the national or even the regional food supply^1,2^. Smallholder farm households are also highly diverse^3^, varying in land area, amount of livestock present, crops grown, and farm management strategies. The importance of understanding the diversity and dynamics of rural households is increasingly crucial, given the diverse effects of global changes in climate, population growth, urbanization, and food demand^4,5^.

Achieving the sustainable development goals (in particular the goals of no poverty and zero hunger, but others too) requires more intensified sustainable food production and development of rural economies. Targeted investment to make progress in agricultural development requires understanding the links between farming practices, livelihood practices, and the effects on farm performance and household welfare. Reliable indicators at farm-household level of both farm performance and household welfare are therefore needed to better understand and model these linkages, and to inform the design and implementation of interventions by governments, donors, and international agencies, across a wide range of differing geographies and socio-economic dimensions^6^.

The lack of standardization of agricultural household surveys, especially in international ‘agriculture for development’ research, has resulted in a proliferation of survey tools and indicators leading to datasets which are often badly documented, incoherent, and with limited interoperability. An example of the consequences of this situation is the study of Frelat *et al*.^3^, which brought together a series of different household survey datasets, but had a hard time defining a common indicator of food security that could be quantified across all these datasets. The current state of affairs limits our ability to compare outcomes across studies and to draw general conclusions on the effectiveness of interventions and the trade-offs between outcomes, which may be shaped by household structure, farm management and the wider social-environmental context^3,5^. Efforts like the CGIAR’s Big Data Platform have also recognized this situation, and try to define common layouts for household surveys and sets of ontologies underpinning the information to be collected in household surveys^7^.

**Fig. 1:**
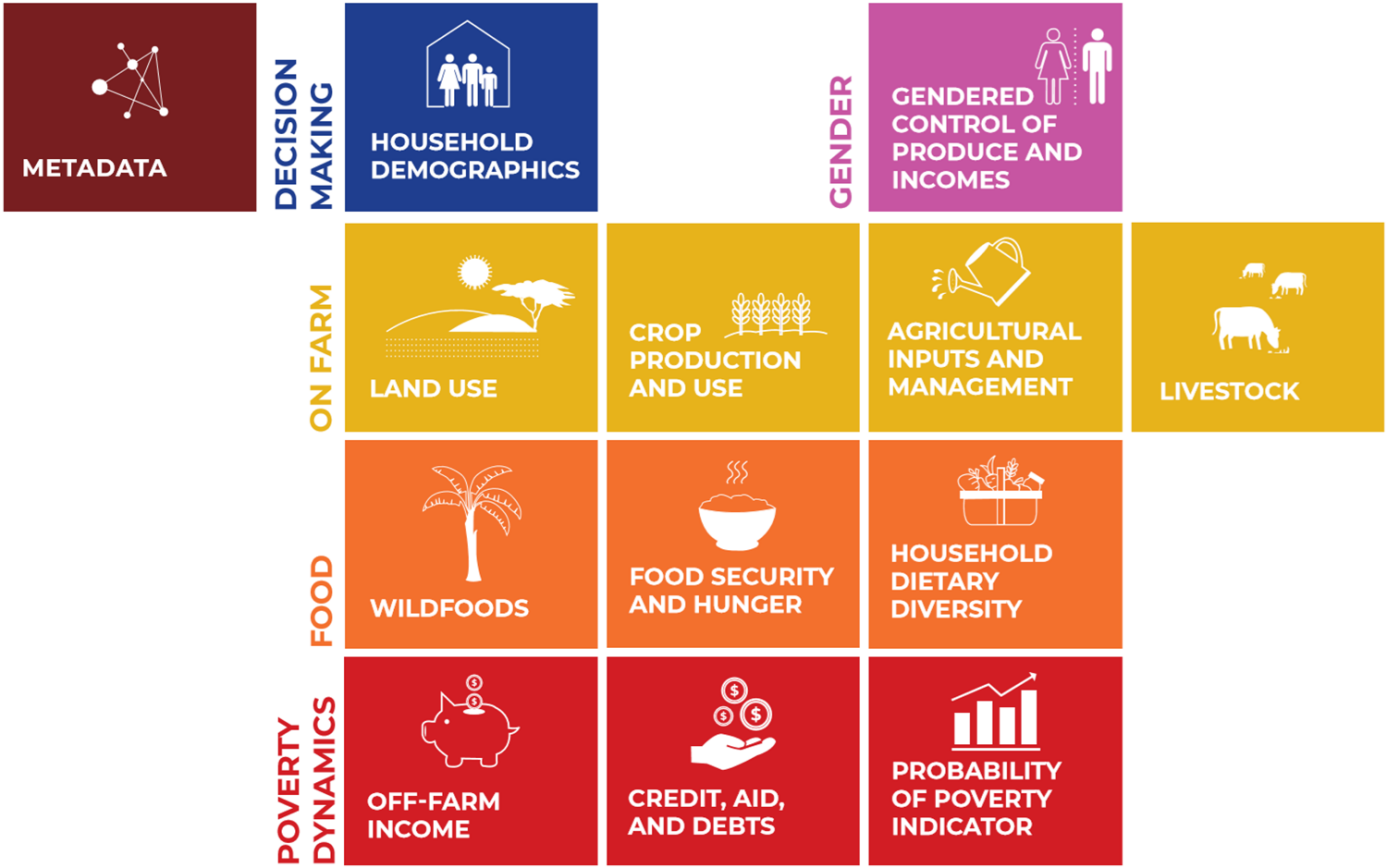
Key indicator groups (modules) generated by all RHoMIS applications.

In contrast, RHoMIS (Rural Household Multiple Indicator Survey; www.rhomis.org) is a standardized household survey approach designed to rapidly characterise a series of key indicators across the spectrum of agricultural production and off farm activities, alongside market integration, nutrition, food security, poverty and greenhouse gas (GHG) emissions^8^ (Fig. 1). It includes a modular survey tool which takes 40–60 minutes to administer per household, a digital platform to store and aggregate incoming data as well analysis code to quantify indicators and visualize results. Optional modules can be bolted-on. The tool has been systematically designed to enable the quantification of interactions between different components and outcomes of agricultural systems, including productivity, and human welfare at the farm and household level, and it has been widely adopted by research organisations and development partners^8^. Such a streamlined, modular approach has resulted in a strong reduction in costs^9^ compared to traditional households surveys in the field (which in other approaches typically take 2–3 hours per household^10^) and of the subsequent data analysis and reporting^11^.

A harmonized dataset has been developed from all the applications of RHoMIS that took place during the years 2015, 2016, 2017 and the first three months of 2018, resulting in a dataset collected from 13,310 farm households across 21 low- and middle-income countries. The overall database (available at the Harvard Dataverse RHoMIS data repository) consists of the raw data (the 758 variables mentioned above; see subdirectory ‘data to share\rhomis_full_data.csv’) and 41 indicators calculated based on the information provided by these variables (see subdirectory ‘data to share\rhomis_indicators.csv’). The raw data and indicators have already been used for a wide range of studies at site level^8,12–17^, for regional analyses^18,19^, and for continental analysis^20^. Different aspects of smallholder households have been analysed, including gender equity^19,21^, dietary diversity^17,18^, nutritional gaps^20^, poverty and GHG emissions in relation to production intensification^8^, subsistence- versus market-orientated strategies^20^, and on-farm vs. off-farm activities^20^. RHoMIS is an on-going initiative, and we welcome interested parties to the community of practice (see www.rhomis.org for up-to-date information and downloadable survey questionnaires). Records continue to be submitted to the central data repository: in the latter part of 2018 more than 10,000 households were additionally interviewed, and their information added to the database. Further releases will be made public in the near future.

## Methods

### Basic characteristics and geographic coverage

**Fig. 2:**
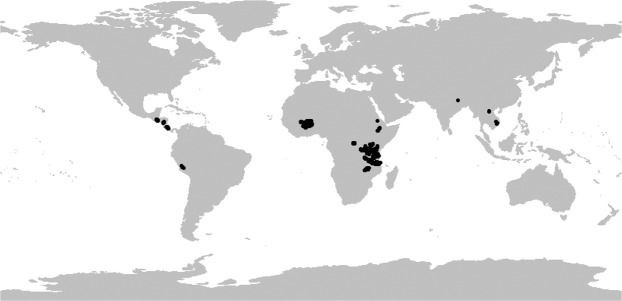
Geographical location of the observations included in the RHoMIS dataset.

The countries in which survey data included in the current database were collected, are summarized in the Online-only Table 1, together with key metadata: the research or development project in which the survey was applied, the lead organisation implementing the survey, the number of households surveyed, and a brief summary of the sampling strategy. The locations of the surveyed households are shown in Fig. 2, demonstrating the geographical breadth of the dataset. The dataset also includes detailed documentation and metadata (‘MetaData RHoMIS survey applications.docx’), describing the aims of each project in which RHoMIS was applied, the sampling strategy used, and additional data collected outside the core set of RHoMIS variables (i.e., topics beyond those presented in Fig. 1). These additional, project specific data that are outside the core RHoMIS variables but are available on request from the corresponding author of this manuscript, always in consultation with the representative of the organisation which executed the RHoMIS application in the field.

The total number of households in the data set is 13,310. The data are all based on single cross-sectional survey applications. In most applications, households were chosen randomly within the sites in which the different implementing projects were working, but some sampling strategies were aimed at evaluations of project interventions (see Online-only Table 1, sampling strategy). Three country-wide applications are available in the current database: two led by the iNGO TreeAID in Burkina Faso, where households were selected across the major agro-ecological zones of the country, and one led by the International Livestock Research Institute in Tanzania that sampled cattle owning households, randomly selected from animal health service records. As is clear from Online-only Table 1, the sample sizes differ per application. It is also of key importance to refer to the metadata information file to assess the representativeness of each of the RHoMIS applications. In previous analyses that aimed for statistical inferences valid for farm type, households were grouped by their farm characteristics^20^, or if an analysis aimed for spatial representativeness, observations were spatially clustered and resampled or weighted by the local population (e.g. village) densities^17^. The varied sampling procedures followed by the individual applications make it essential to weight and/or re-sample households in any analysis making use of this combined RHoMIS dataset for valid statistical inferences.

### The questionnaire

The RHoMIS questionnaire is a set of carefully, expertly designed modules that are administered digitally using the ODK software platform (https://opendatakit.org/)^8^. The survey is designed to be both flexible enough to suit local contexts and sufficiently standardised to permit rapid deployment, analysis and comparison between multiple sites, without the need for costly post-survey harmonization. The data package that is made available consists of two parts: the dataset itself (containing the raw data and the indicator results) and secondly, the series of documents and analysis code files underpinning the raw data collection effort and the subsequent indicator quantification. We have made the survey available in easily readable pdf format (‘RHoMIS for printing_v1.3.pdf’). The questionnaires and their variable names have been linked to a supporting set of data extraction and analysis tools written in R (https://cran.r-project.org/), also included in the package. The majority of questions in the survey are used for the estimation of a series of pre-defined indicators that include:

- The Household Food Insecurity of Access Scale^22^ for measuring the frequency and severity of hunger (this indicator in more recent applications of RHoMIS is replaced by the FIES – Food Insecurity Experience Scale indicator; http://www.fao.org/in-action/voices-of-the-hungry/background/en/).
- The Household Dietary Diversity Score^23^, providing an indicator of household dietary adequacy; this indicator was adapted to cover both the bad and good seasons
- The Probability of Poverty Index^24,25^, an asset-based scoring system to estimate the likelihood that a household is in poverty.
- The Potential Food Availability indicator for quantifying the ability of a household to feed itself through both on-farm and off-farm activities^3,4^.

These indicators are combined with a comprehensive inventory of agricultural crops and livestock production characteristics, including yields, the use of products (consumption, sale, etc.), product sale prices, input use, and an assessment of off-farm incomes. The data captured in the RHoMIS tool place the farm household along a continuum of household and farm characteristics, performance indicators, and welfare indicators (see Fig. 1), enabling in-depth analyses of individual indicators, but also integrative analyses of how indicators co-vary and how on-farm and off-farm livelihood strategies correlate to food security, poverty, and dietary diversity. The questionnaire is organised into seven sections wherein respondents are asked to provide information on the previous 12 months’ farming and non-farming activities (Table 1). We calculated the indicator values using custom code, available under the subdirectory ‘R scripts’ at the Harvard Dataverse RHoMIS data repository. The setup of the code is explained in detail in ‘RHoMIS Data Processing Doc.docx’, and a pdf and an excel ODK definition file explaining each variable, are also supplied with the data.

**Table 1:** General overview of the information (captured by the 758 variables shared in the dataset) captured, organised by indicator group (see Fig. 1) in RHOMIS.

Indicator Group	Questions
Metadata	Informed consent; location of the survey (village, district, region, country); project beneficiary; local monetary unit; GPS coordinates of where the interview took place
Household demographics	Respondent sex; whether respondent is household head; age of respondent; type of household (single female; single male; couple; couple - spouse living away); age and sex of household members
Land use/tenure	Total land area cultivated; amount of land owned; access to common land; land rented out/in
Crop production and use	Crops/vegetables/fruits/trees grown, area grown; production per year/season; use of products (sold, consumed, stored, processed, given away); price of crop products; use of crop residues
Agricultural inputs and management	Use of mineral fertilizer, manure, pesticides; irrigation: type, area and/or amount used; intercropping; agroforestry; medicine use for livestock
Livestock	Livestock species kept on farm; production per year/season; use of products (sold, consumed, stored, etc.); price of livestock products
Wild foods	Relative importance of wild foods for food security; months of the year collected; most important wild food items
Food security and hunger	9 standard questions of Hunger and Food Insecurity Access Scale (HFIAS); number of months with hunger; identification of worst and best month in terms of food security
Household dietary diversity	10 food groups of the Minimum Dietary Diversity Score for Women in the worst, best and last month; source of each food group (farm-based, purchased, gathered or a combination of sources)
Off-farm income	Relative importance of off-farm income in overall income; use of income (reinvestment in farm, household (non-food) expenses, food purchase)
Credit, aid and debts	Household access to credit; dependency on/receipts of aid in the last 12 months, from institutions or neighbours; household debt
Probability of Poverty Index (PPI)	10 standard questions, country-specific (see https://www.povertyindex.org/)
Gendered control of produce and income	Per produce and income item: who takes decisions on sale, spending, and consumption

The data collection efforts conformed with the principles of the 1964 WMA declaration of Helsinki. Ethical approvals for the survey applications was obtained by the internal ethical review committees of the different institutes (e.g. the Internal Review Ethics Committee (IREC) of the International Livestock Research Institute) or for those partners without an internal ethical committee, by ethical evaluation by the senior management at each organization after careful evaluation of the content, methodology, and with oral informed consent statement built-in to the survey. Survey participants were not particularly vulnerable, data was processed in anonymized form, and survey participants had the possibility to skip questions. Explicit oral informed consent was obtained from all survey participants prior to survey enumeration and documented as the opening question in the RHoMIS survey upon informing survey participants of the study’s purpose. If consent was denied, survey enumeration was terminated. Permission for obtaining oral rather than written consent from survey respondents was granted by the Internal Review Ethics Committee (IREC) of the International Livestock Research Institute, implementing research organizations and local agricultural officers, given literacy limitations among the target populations.

#### Data processing and indicator calculations

**Fig. 3:**
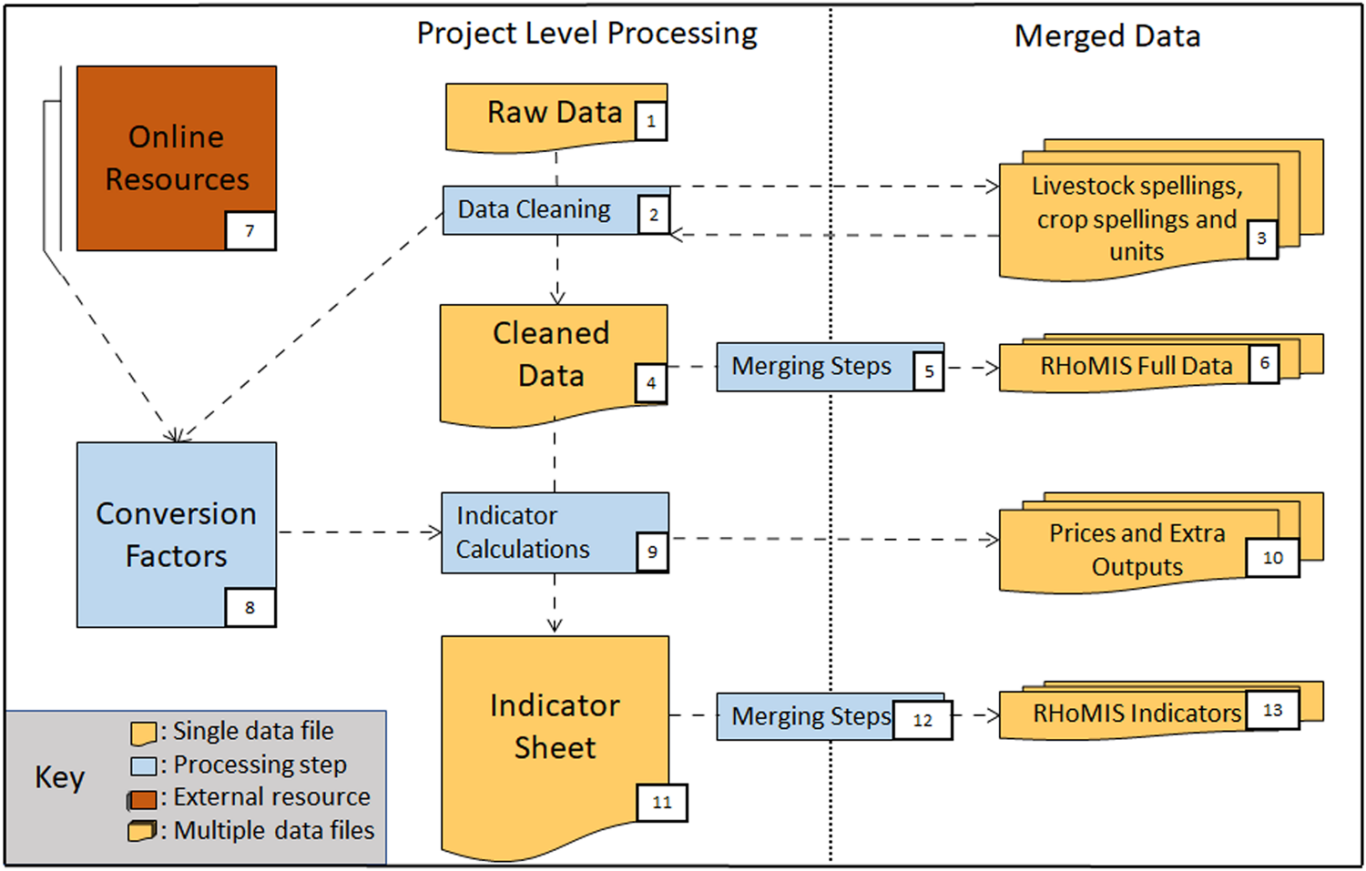
Overview of the data processing and indicator calculation steps.

A standard set of scripts has been created in the R software environment and used to process the raw data provided in this dataset, and for the calculation of the various indicators. An overview of the data processing and indicator calculations is given in Fig. 3, differentiating between outside information used (e.g. energy content of food items, in dark brown), the different data products produced that are included in this dataset (in light brown) and the processing steps (in blue). We now explain the procedure in more detail. The starting point are the ‘raw collected’ data. We have limited the data cleaning step (step 2) to only correcting obviously unrealistic values, such as when it was clear that the recorded value in combination with the reported unit generated an impossible indicator value (say 1.5 *kg* of maize yield from a 1 ha field; here it is clear that the unit should be *tons* of maize; or similarly if a production of 3,000 *tons* from a 1 ha field is reported, it is clear that the unit should be *kg*). However, the most important step in the cleaning process was unifying crop names, livestock species names, and the crop and livestock product names (step 2 & 3 in Fig. 3). This cleaned data from all survey implementations was then merged into a single raw data csv-file, supplied in the data package (step 6 in Fig. 3).

Conversion factors use in the indicator calculations, for example area units, monetary units, or energetic content of foods, are based on literature resources (step 7 in Fig. 3). Within the calculations of some indicators, e.g. income, value of farm production, or crop and livestock productivity, intermediate output variables were also generated: farm gate prices of farm products, and crop and livestock production values per individual crop or livestock species (step10 in Fig. 3). These are included in the data package. The final product of the indicator calculations is the merged RHoMIS indicator results file, step 13 in Fig. 3. The annotated R code, for performing and documenting all the processing and calculation steps illustrated in Fig. 3, is also supplied. In addition, the individual indicator calculations are explained in detail in the ‘Explanations_of_Calculations_and_Outputs.xlsx’ file.

With the current data management system we follow the FAIR principles: Findability, Accessibility, Interoperability, and Reusability^26^. By using standardized data-labelling and data processing approaches across all current and future RHoMIS applications we follow the principle of ‘assisting machines in their discovery and exploration of data through application of more generalized interoperability technologies and standards at the data/repository level’. The FAIR principles are adhered to via (i) the extended metadata and documentation available at project and survey level (e.g. ‘MetaData RHoMIS survey applications.docx’); (ii) the publicly available survey and processing software; as well as (iii) the standardized approach of the core RHoMIS survey.

## Data Records

The RHoMIS data^27^ can be found at Harvard Dataverse, 10.7910/DVN/9M6EHS.

All 758 variables of the survey data are described in the file ‘Raw Data code book.xlsx’. The variables included in indicator results file are described in ‘RHoMIS Data Processing Doc.docx’. We have also included the RHoMIS survey in ODK definition and pdf format to further facilitate the interpretation of the variable names. The RHoMIS survey is continuously updated; the newest version can be found at www.rhomis.org.

## Technical Validation

Each RHoMIS survey application included in the data set has undergone a series of standard data quality evaluation steps to assess the overall quality of the data collected. This did not lead to removal of individual observations, we prefer that the user of the data can make their owned informed decisions on this topic. The validation information supplied here is only advice on how the data user might be able to check the quality of the data they want to use and to be able to base decisions on a systematic approach if he or she does decide to remove observations or data points. Note that we used the validation approach (described below) to evaluate each application made available in the current dataset, and that for each application no more than 25% of the data were found to need more attention, which compared to other household survey tools is a good score^11^.

Before validation, two key quality filters were applied, correcting some of the observations. One is the earlier mentioned ‘correcting obviously unrealistic values’, see the text accompanying Fig. 3 for explanation. The second is correction of farm gate price values for the different types of farm produce, which is used in the calculation of several RHoMIS reported indicators. Especially when there are low numbers of observations of a certain type of farm produce, reported price values can be quite extreme values, even when median values are calculated across the dataset of the survey application. We therefore defined ranges of prices (price reported plus or minus 100%) for each commodity based on FAOSTAT data to especially avoid unrealistically high prices which would affect some of the indicators strongly.

**Fig. 4:**
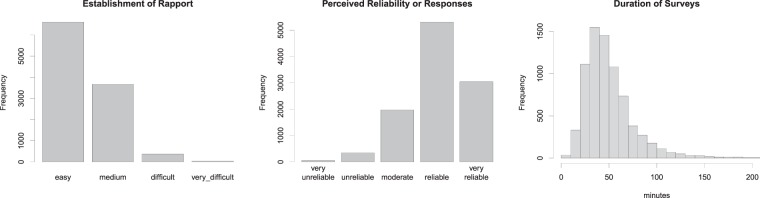
Overview of the three key aspects considered in step 1 of the data quality evaluation (for explanation see text): subjective enumerator evaluation of data quality through ‘establish rapport’, ‘perceived reliability’, and survey duration.

The validation process entailed three steps. In step 1 consists of subjective evaluation by the enumerators themselves. These questions are ‘In your opinion, how easily did you establish rapport with the respondent?’ (with possible answers: ‘Easily’, ‘OK’, ‘Difficult’ and ‘Very difficult’) and ‘How reliable do you think these answers are? Consider the accuracy and willingness to answer.’ (with possible answers: ‘Very reliable’, ‘Reliable’, ‘OK’, ‘Occasional doubts’ and ‘Regular or serious doubts’). This information, in combination with the total survey duration (based on start and end time of survey implementation), provides insight on the overall reliability of the information recorded. For example, if the survey duration is extremely long (e.g. beyond 2 h) it is also likely that data quality will be questionable. Example results of these quality indicators are given in Fig. 4, showing the typical distribution of enumerator observed reliability and survey length of survey. This may serve as a useful norm for the initial quality assessment of new RHoMIS applications. The information collected in individual survey applications where the reliability answers show low scores, or where many survey records show abnormally short or long durations, can be more thoroughly investigated or even rejected.

**Fig. 5:**
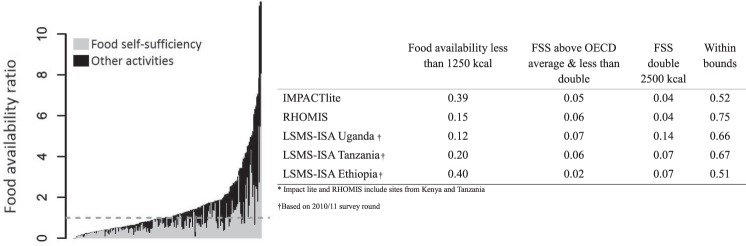
Step 2 of the data quality evaluation: the identification of systematic under- or over-estimation of production and consumption values based on food self-sufficiency and potential food availability, presented as the relative number of observations. RHoMIS results are compared with results obtained by other survey instruments (ImpactLite and the Living Standards Measurement Survey – Integrated Survey on Agriculture (LSMS-ISA)) in the same countries. Reported elsewhere in detail^11^.

In step 2 we follow the food availability/self-sufficiency approach^3,11^ to evaluate the overall reliability of the data on production and consumption of farm produce. Calculated livestock productivity and crop yield values are compared to realistic ranges normally encountered in smallholder systems in the agro-ecosystem of interest^28^. Because the plausible value ranges for these checks are still large, we also scrutinise composite indicators that combine information from a number of survey variables. The two composite indicators we examine for quality assessment are food self-sufficiency and potential food availability^3^. The food availability indicator represents the total food energy potentially available daily per household member (adjusted to adult male equivalent calorie demand), and is calculated from the reported consumption of farm products, from cash sales of farm products, and from off-farm activities, whereby all income is converted to a calorific value based on the cost of a local staple crop. Results of these calculations can be used to assess the data quality of information on crop and livestock production, sales and consumption as well as off-farm income. Two problems with this composite indicator are commonly encountered. First, a considerable number of household records at the lower end of the food availability scale appear to suggest an underestimation of calorie availability, suggesting an extreme level of starvation. This may be a true representation of some households, but it can also be an indication of missing information on income or food consumption. Second, a substantial number of households can also show a substantial over-estimation of consumption of crop and livestock products – indicating possible problems with yield, consumption or household size estimates. We set a lower bound threshold for food availability at 1,250 kilocalories (kcal) per male adult equivalent per day, which is below the basal metabolic rate for adult males (approximately 1,590 kcal for a 60 kg male) and adolescents (1,360 kcal for a 40 kg adolescent male). We set an upper bound for food self-sufficiency (i.e. consumption only) at: (a) 3,500 kcal per male adult equivalent per day, representing the average intake of developed nations^23^; and (b) 5,000 kcal, which is double the approximate requirement for an adult male. Observations which fall outside these bounds (e.g. Fig. 5) are used to examine the overall reliability of the survey application and can be used to identify individual survey applications where the data do not appear reliable from a composite perspective. Typically, between 10 and 25% of the records within the total survey sample of an application site may show questionable values of these indicators. These performance values are representative for the uncertainty encountered in recall based cross-sectional farm household surveys, and actually better than the performance of two other widely used sources of farm household information (Fig. 5). All sites included in the databases published in this article fall within this performance range. Further trust in these findings can be developed by triangulating these results with other indicators of food security included in the RHoMIS surveys, for example HFIAS, dietary diversity and the number of months with hunger (see also step 3).

**Fig. 6:**
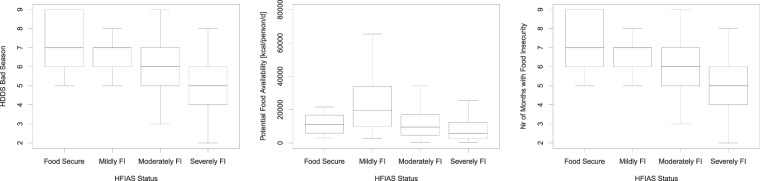
Relationships encountered between the household dietary diversity score in the bad season (HDDS Bad Season), potential food availability, the number of months with food insecurity, and the Hunger and Food Insecurity Access Scale (HFIAS) status, based on the 2016 Kenya Wote dataset.

In step 3 we cross-checked relationships between different food security indicators. The information collected by RHoMIS underpins 4 different indicators of food security (HFIAS, dietary diversity, number of months with hunger, and potential food availability). Even though these indicators capture different aspects of food security, we do expect strong correlation between them. Figure 6 provides examples of the typical relationships encountered between these variables. A strong deviation of the relationships found in a new RHoMIS application from these typical relationships may necessitate a deeper investigation, and might indicate questionable data quality in one or more of these indicators.

## Usage Notes

The objective of RHoMIS is to gather information on the common variables of interest in all agricultural development research, but not to go too deep into any one topic. The overall strategy of RHoMIS is to collect data which permits an overview of the farming system and the main livelihood activities. Based on this information we can identify farm level constraints, deficiencies or successes, and sift meaning from the high degree of variation observed amongst smallholder households. This is in contrast to the design of many impact assessment studies, which collect data on a narrow topic but at a higher resolution, thus permitting evaluation of that specific topic, but limiting the ability to assess the over-arching farming system and rural livelihoods. This dataset can be used to investigate the characteristics of agricultural systems in low- and middle-income countries at one point in time. On- and off-farm strategies can be analysed in relation to a series of welfare indicators representing food security, poverty and gender equity. This can be done both at individual household level, to disentangle the livelihood strategies of specific household types, but also at population level to determine more generic patterns and investigate equity aspects. We emphasize that the results of such analysis should be interpreted within the context of the data set, considering the potential biases and limitations described in this paper. It is especially important that users note the varied sampling procedures followed in the different RHoMIS applications, and recognise that it essential to weight and/or re-sample the households in any analysis before making any across-site statistical inferences. See the Methods section for more details.

## Online-only Table

**Online-only Table 1:** RHoMIS applications included in this data set.

Country (site/region)	Lead Institute	Year	Households surveyed	Short description of the sampling strategy
Burkina Faso	International Livestock Research Institute (ILRI)	2015	200	Fifty households were randomly sampled from one village in each of the four project regions.
Burkina Faso	International Livestock Research Institute (ILRI)	2016	400	Fifty households were randomly sampled from 4 villages in each of the two regions.
Burkina Faso	TreeAID	2018	1030	Smallholder producers were selected randomly from 50 villages across Burkina Faso. The villages were selected according to already established relationships with partner organisations.
Burkina Faso	TreeAID	2018	1270	Smallholder producers were selected randomly from 60 villages across Burkina Faso. The villages were selected according to already established relationships with partner organisations.
Burundi	International Livestock Research Institute (ILRI) - International Institute of Tropical Agriculture	2018	333	Households were interviewed in the regions of Cibitoke and Gitega. In both regions households were randomly sampled from the available village lists obtained from the village elderly.
Cambodia	International Center for Tropical Agriculture (CIAT)	2016	631	Respondents were randomly selected from village and commune lists obtained from local authorities by national partners.
Costa Rica	Bioversity International	2017	204	Respondents were selected through random selection in the Caribbean humid tropic region of Costa Rica based on lists of producers available at the ministry.
Democratic Republic of Congo	World Agroforestry (ICRAF)	2017	413	Households were interviewed along three of the four borders surrounding the Yangambi Biosphere Reserve. The two-stage sampling procedure included purposeful sampling (used to equitably select villages along the borders according to population sizes) and random sampling within villages to select households.
Democratic Republic of Congo	International Institute of Tropical Agriculture (IITA) - International Livestock Research Institute (ILRI)	2017	438	Households were interviewed in the northern and southern region of Bukavu. Households were randomly sampled from the available village lists obtained from the village elderly.
Ethiopia	TreeAID	2017	302	Households were interviewed in the region north-east of Meki. Subsistence oriented households were randomly sampled from the available village lists obtained from the village elderly.
Ethiopia	Bioversity International	2017	249	Households were randomly sampled from beneficiary villages involved in an ongoing research project led by Bioversity International by sampling constant number of smallholder farmers per village.
Ethiopia	TreeAID	2018	405	Smallholder producers were selected randomly from 8 villages across the Bosena Werda area in the central highlands in Ethiopia. The villages were selected according to already established relationships with partner organisations.
Ghana	TreeAID	2017	223	Informants were selected randomly from 26 villages within the project area, where informants were either project beneficiaries (101 households) or were not beneficiaries – a ‘control group’ (122 households). The villages were selected according to already established relationships with partner organisations.
Ghana	TreeAID	2018	391	Smallholder producers were selected randomly from 10 villages across the Navrongo region in northern Ghana. The villages were selected according to already established relationships with partner organisations.
India	International Livestock Research Institute (ILRI)	2016	160	In an earlier household survey application in 2012 200 households were randomly sampled from a 10 by 10 km block. In this RHoMIS application 160 households were randomly selected from these original 200.
Kenya (Central-West)	International Livestock Research Institute (ILRI)	2016	160	In an earlier household survey application in 2012 200 households were randomly sampled from a 10 by 10 km block. In this RHoMIS application 160 households were randomly selected from these original 200.
Kenya (South)	International Livestock Research Institute (ILRI)	2016	160	In an earlier household survey application in 2012 200 households were randomly sampled from a 10 by 10 km block. In this RHoMIS application 160 households were randomly selected from these original 200.
Kenya	Bioversity International	2017	316	Households were randomly sampled from beneficiary villages involved in an ongoing research project led by Bioversity International by sampling constant number of smallholder farmers per village.
Kenya	World Agroforestry (ICRAF)	2017	385	Households were interviewed in the Kitui and Baringo regions, two regions different in agroecological zone and livelihood strategies. In each region, four divisions were purposefully selected based on population density. Within each division, two sub-locations were purposefully selected to capture economic and geographic heterogeneity. Forty to sixty households were randomly selected to participate in each sublocation.
Kenya	International Livestock Research Institute (ILRI)	2017	169	Respondents were selected through a two-stage, stratified cluster random sampling, one at the ward level and the other at the household level for each county. A random sample of wards is selected with probability proportional to the size of the population and number of households in each ward relative to the total population in all the wards in the counties. The wards are selected randomly from a list of all wards in the counties. The counties represent the strata and when a ward is selected, all the households within the ward are also automatically selected, as in cluster sampling. For this study, a total of 169 households were selected as the representative sample from all the six counties in Western Kenya.
Laos	International Center for Tropical Agriculture (CIAT)	2016	365	Respondents were randomly selected from village and commune lists obtained from local authorities by national partners.
Malawi	International Maize and Wheat Improvement Centre (CIMMYT)	2015	160	The register of farm members of the National Smallholder Farmers Association of Malawi was used to randomly select 80 Households each in the + HHM (households exposed to the Household Methodology; this was in Lilongwe North) and -HHM sites (this was in Lilongwe South), respectively.
Mali	International Livestock Research Institute (ILRI)	2015	200	Fifty households were randomly sampled from one village in each of the four project regions.
Mali	TreeAID	2017	363	Households were selected randomly from 21 villages within the project area. The villages were selected according to already established relationships with partner organisations.
Nicaragua	Bioversity International	2018	145	In the area of La Dalia 145 households were randomly chosen based on existing village lists within the climate smart village run by the CCAFS program.
Peru	Grupo Yanapai - Colorado State University	2018	174	Households were interviewed in Huancayo, Acobamba and Ocros. In all three sites households were randomly sampled based on village lists available from census information.
Tanzania	International Livestock Research Institute (ILRI)	2015	150	In an earlier household survey application in 2012 200 households were randomly sampled from a 10 by 10 km block. In this RHoMIS application 150 households were randomly selected from these original 200.
Tanzania	International Livestock Research Institute (ILRI)	2017	996	Respondents were randomly selected from village and commune lists of cattle owners obtained from animal health service records.
Tanzania	Bioversity International	2017	528	Forty-four villages were randomly selected from administrative village lists for data collection (20 villages each in Mtwara and Lindi, and 4 villages in Tunduru). At each village, 12 farming households were randomly sampled from lists provided by local extension officers.
Tanzania	World Agroforestry	2018	841	Households were interviewed in the regions of Tabora, Dodoma, Mafinga and Zanzibar. In all four sites households were randomly sampled based on village lists obtained from the village elderly.
Trifinio (Guatemala, El Salvador, Honduras)	Bioversity International	2015	300	Across the Trifinio area, 300 households were sampled from lists provided by organizations that provide development services to these communities. Communities were sampled as those where Farmer Field Schools could be organized. From each selected community, 2 households were randomly sampled for the RHoMIS survey, balanced across communities.
Uganda	Wageningen University	2017	130	In an earlier household survey application in 2012 200 households were randomly sampled from a 10 by 10 km block. In this RHoMIS application 130 households were randomly selected from these original 200.
Vietnam	International Center for Tropical Agriculture (CIAT)	2016	310	Respondents were randomly selected from village and commune lists obtained from local authorities by national partners.
Zambia	World Agroforestry (ICRAF)	2017	600	Households were interviewed in the regions of Petauke, Katete and Chipata. In all three sites villages and households were randomly sampled based on village lists obtained from NGO collaborators.
